# DNA methylomes and transcriptomes analysis reveal implication of host DNA methylation machinery in BmNPV proliferation in *Bombyx mori*

**DOI:** 10.1186/s12864-019-6146-7

**Published:** 2019-10-15

**Authors:** Haoling Huang, Ping Wu, Shaolun Zhang, Qi Shang, Haotong Yin, Qirui Hou, Jinbo Zhong, Xijie Guo

**Affiliations:** 1 Sericultural Research Institute, Jiangsu University of Science and Technology, Zhenjiang, 212018 China; 2 The Key Laboratory of Silkworm and Mulberry Genetic Improvement, Ministry of Agriculture and Rural Affairs, Sericultural Research Institute, Chinese Academy of Agricultural Sciences, Zhenjiang, 212018 China; 3 Quality inspection center for sericultural products, Ministry of Agriculture and Rural Affairs, Zhenjiang, 212018 China

**Keywords:** *Bombyx mori*, Epigenetic regulation, BmNPV, DNA methylation, Host-virus interaction, Insect immune response, inhibitor of apoptosis

## Abstract

**Background:**

*Bombyx mori* nucleopolyhedrosis virus (BmNPV) is a major pathogen that threatens the sustainability of the sericultural industry. DNA methylation is a widespread gene regulation mode in epigenetics, which plays an important role in host immune response. Until now, little has been known about epigenetic regulation on virus diseases in insects. This study aims to explore the role of DNA methylation in BmNPV proliferation.

**Results:**

Inhibiting DNA methyltransferase (DNMT) activity of silkworm can suppress BmNPV replication. The integrated analysis of transcriptomes and DNA methylomes in silkworm midguts infected with or without BmNPV showed that both the expression pattern of transcriptome and DNA methylation pattern are changed significantly upon BmNPV infection. A total of 241 differentially methylated regions (DMRs) were observed in BmNPV infected midguts, among which, 126 DMRs were hyper-methylated and 115 DMRs were hypo-methylated. Significant differences in both mRNA transcript level and DNA methylated levels were found in 26 genes. BS-PCR validated the hypermethylation of *BGIBMGA014008*, a structural maintenance of chromosomes protein gene in the BmNPV-infected midgut. In addition, DNMT inhibition reduced the expression of inhibitor of apoptosis family genes, *iap1* from BmNPV*, Bmiap2, BmSurvivin1* and *BmSurvivin2*.

**Conclusion:**

Our results indicate that DNA methylation plays positive roles in BmNPV proliferation and loss of DNMT activity could induce the apoptosis of infected cells to suppress BmNPV proliferation. Our results may provide a new idea and research direction for the molecular mechanism on insect-virus interaction.

## Background

DNA methylation, as one of the important epigenetic regulations, occurs in both eukaryotes and prokaryotes. Accumulating evidences have linked epigenetic effects to various biological processes including gene regulation, development, nutrigenomics, tumorigenesis, and DNA repair in mammals and plants [1–3]. Recently, the roles of DNA methylation in host immune responses have attracted increasing attention. Many studies have demonstrated that viruses, especially DNA tumor viruses, could induce aberrant DNA methylation of host genome to suppress the host immune responses and evade antiviral immunity [4–6]. In addition, viruses could modulate host DNA methyltransferase (DNMT) for epigenetic dysregulation of immune-related gene expression in host cells [7, 8]. Interestingly, demethylation treatment of cancer cells could activate the virus RNA transcription from dormant endogenous retroviruses and trigger antiviral signaling [9, 10].

Although comparing to mammals, the researches on DNA methylation in insects are relatively lagging behind, with the improvement and innovation of DNA methylated research methods, especially for the rapid progress in large-scale sequencing technology, some progresses and achievements on DNA methylation in insects have been made in recent years. Based on whole genome bisulfite sequencing (WGBS), insects such as *Drosophila melanogaster, Tribolium castaneum*, *Bombyx mori* and *Nasonia Vitripennis* [11–14] have proven that DNA methylation in insect genome does exist and the maintenance and regulation mechanism of DNA methylation system in insects may greatly differ from that of mammals and plants [15–17]. The function of DNA methylation generally associates with the evolution, aging, memory and regulation of caste determination in honey bees and in ants [18–21].

Until now, researches on the function of DNA methylation in insect’s immune responses are very limited. It is reported the genome wide-patterns of DNA methylation in the *Aedes aegypti* are disrupted with the infection of a virulent strain of *Wolbachia* [22]. Three different strains of *Metarhizium anisopliae* caused the differential expression of *dnmt* genes in the larvae of *Galleria mellonella* infected in a natural manner, suggesting that DNA methylation may play roles in the immune response of insects against parasitic fungi [17]. In *Bombyx mori* only two DNMT have been reported, DNMT1 and DNMT2. BmDNMT-1 retained the function as maintenance DNMT [23].. Our previous study found that 27 genes were shown to have both differential expression and differential methylation in the midgut and fat body of *Bombyx mori* cytoplasmic polyhedrosis virus (BmCPV) infected larvae, respectively, indicating that the BmCPV infection-induced expression changes of these genes could be mediated by variations in DNA methylation [24].

*Bombyx mori* nucleopolyhedrosis virus (BmNPV), a circular double-stranded DNA (dsDNA) virus, belongs to the family *Baculoviridae* [25] and is a big challenge for the development and sustainability of the sericultural industry [26]. Early study has found that in *Autographa calfomnica* nuclear polyhedrosis virus (AcNPV), the activity of the p10 gene promoter was severely affected when the 5′-CCGG-3′ site in this promoter was methylated, suggesting that methylation of specific promoter sequences in an insect virus can affect viral gene expression [27]. Up to now, there has been scarce report on epigenetic function on BmNPV infection and immune response in *Bombyx mori*.

In this study, we treated the *Bombyx mori* cells with Zebularine (Zeb), a kind of DNMT inhibitor and found that BmNPV replication is significantly suppressed. Furthermore, genome-wide methylome and transcriptome analyses were carried out to explore the possible role of DNA methylation in silkworm immune response and BmNPV-host interaction.

## Results

### DNA methyltransferase inhibition suppresses the replication of BmNPV

**Fig. 1:**
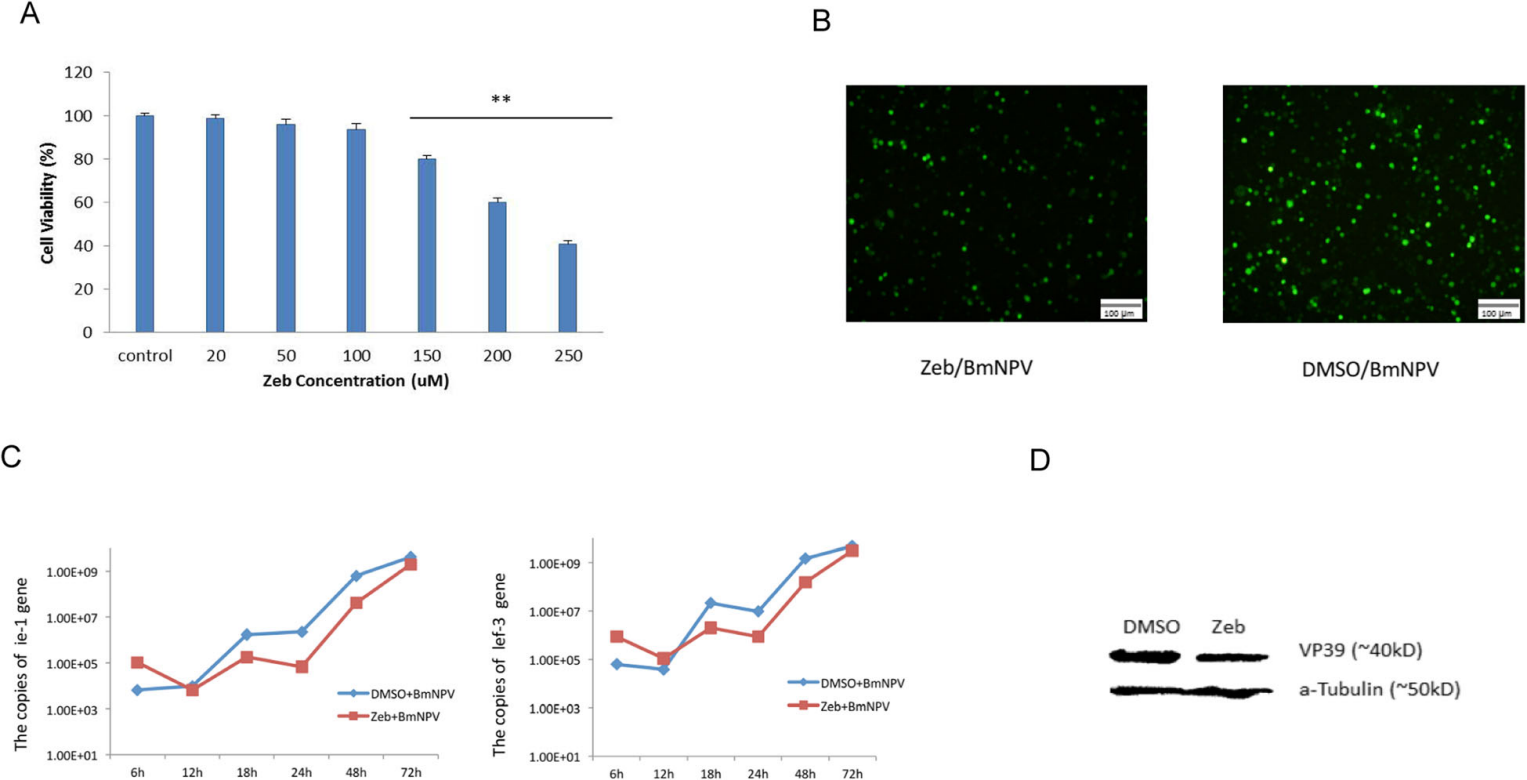
Determination of the cytotoxicity of zebularine and the chemicals effect on BmNPV proliferation. **a** BmN cells were treated with different concentrations of zebularine (20, 50, 100, 150, 200, 250 μM) for 12 h. Cytotoxicity was measured by MTT assay. Cellular cytotoxicity was determined in duplicate and each experiment was repeated three times. ** *p* < 0.01.**b** BmN cells were treated with 100 μM zebularine and infected with recombinant BmNPV BVs containing an *egfp* marker gene (Zeb/BmNPV). DMSO-treated BmN cells with recombinant BmNPV infection were used as the control (DMSO/BmNPV). Fluorescence intensity was observed under fluorescence microscopeat 72 hpi. **c** RNAs were extracted from BmNPV-infected BmN cells treated with zebularine and DMSO, respectively. Absolute qRT-PCR was carried out to analyze the copies of *ie-1* and *lef-3* at different time points. The data were represented as mean ± SD. Three independent experiments with three technical replicates were performed. **d** Western blotting analysis of VP39. Protein samples were from BmNPV-infected BmN cells with zebularine and DMSO, respectively. Western blot analysis for detection of VP39 was performed by an anti-VP39 antibody. a-Tubulin was served as the loading control

Zeb has been well known as an inhibitor of DNMT. We first tested the cytotoxicity of Zeb for BmN cells. BmN cells were treated with different concentrations of Zeb for 12 h followed by MTT assay. The result showed that there were no significant differences in cell survival among 20 μM, 50 μM, 100 μM Zeb-treated cells and control cells (Fig. 1a). The viability of 150 μM treated cells only accounted for 80.17% in control cells. As the Zeb concentration increased to 200 μM, the viability was less than 50% compared to that of control cells. This suggested that less than 100 μM Zeb has no significant harmful effects on BmN cell survival.

In order to examine whether DNA methyltransferase inhibition affects BmNPV replication, BmN cells were then treated with 100 μM Zeb for 12 h and then replaced with a new medium followed by infection of recombinant BmNPV BVs containing an *egfp* marker gene for 72 h. By observing under the fluorescence microscope we observed that both the amount of cells with fluorescence and the fluorescence intensity of the treated group (Zeb/BmNPV) were significantly less than that of the control group (DMSO/BmNPV) (Fig. 1b). Subsequently, qRT-PCR was carried out to detect the expression of BmNPV *ie-1* gene and *lef-3* gene at different time points. Comparing the results of Zeb-treated cells to control cells, we found that the expression level of these two genes were very similar with the tendency of increase at 6 h while decrease at 12 h–48 h in Zeb-treated cells (Fig. 1c). Finally, western blot was performed to assess the Zeb impact on BmNPV replication (Fig. 1d). All of these results suggested that inhibition of DNMT could suppress BmNPV proliferation.

### BmNPV infection alters the transcriptional profiles of silkworm midgut

**Fig. 2:**
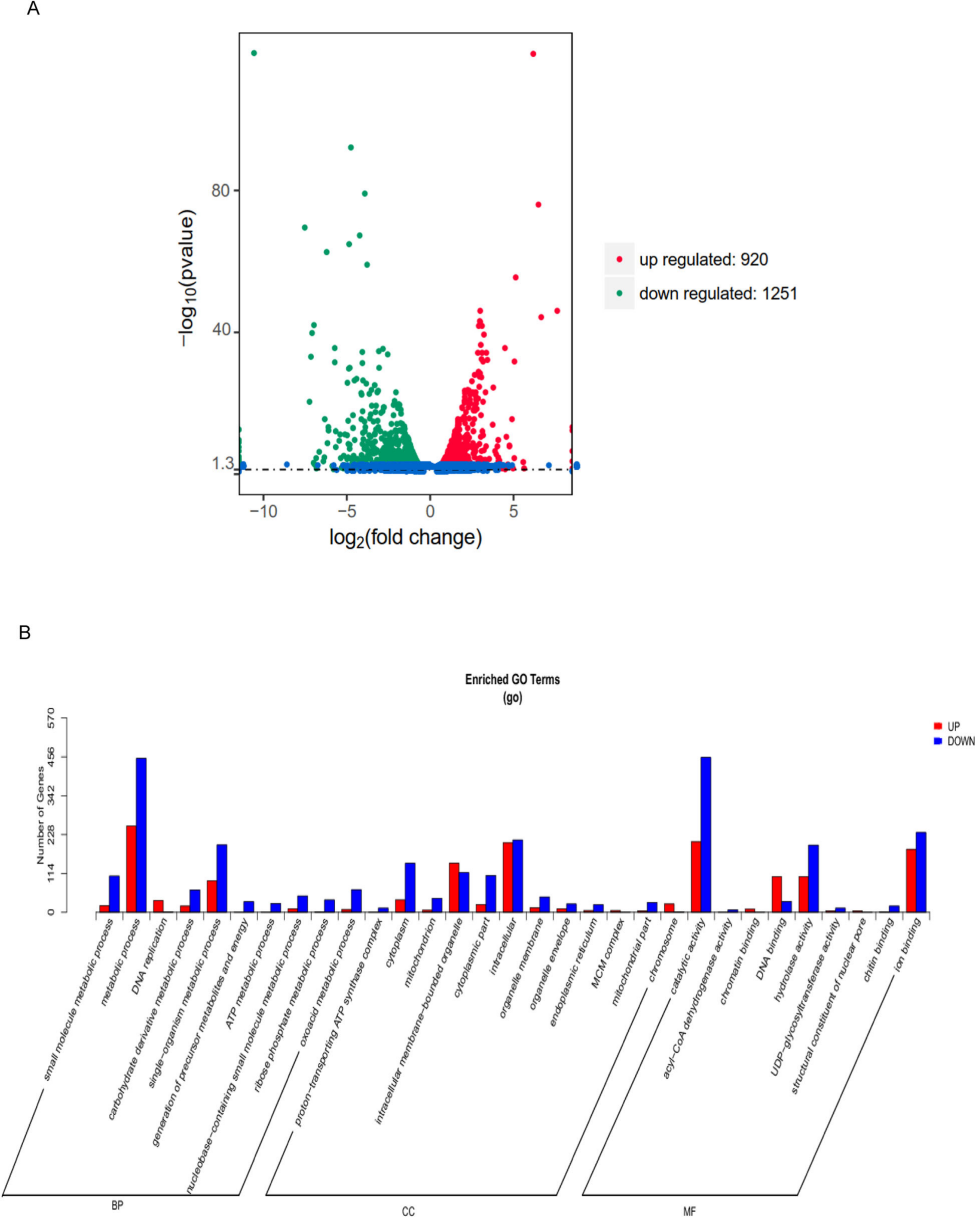
Differentially expressed genes associated with BmNPV infection. **a** The volcano plot of differentially expressed genes. Red points represent up-regulated genes; Green points represent down-regulated genes. Blue points represent non-changed genes. **b.**GO anotation was performed by mapping genes to GO terms in the GO database (http://www.geneontology.org). GO enrichment analysis was conducted by GOseq R package with corrected *p*-value ≤0.05 as a threshold. Gene numbers are listed for each category

By RNA-Seq technology, we analyzed the transcriptional profiles of normal and infected silkworm midgut. More than 43,000,000 clean reads were obtained from each sample and the summary of the sequenced data is shown in Additional file 1: Table S1. The sequence data are deposited in the NCBI Sequence Read Archive database (http://www.ncbi.nlm.nih.gov/sra/) under the accession number PRJNA541379. The number of significantly differentially expressed genes (DEGs) in infected midgut was 2171, with 920 up-regulated genes and 1251 down-regulated genes (Fig. 2a, Additional file 1: Table S2). GO analysis revealed that the down-regulated genes were enriched in ATP metabolic process, cytoplasm, catalytic activity, small molecule metabolic process, etc. Up-regulated genes were enriched in DNA binding, chromosome, DNA replication and so on (Fig. 2b). KEGG pathway analysis showed that up-regulated genes were involved in DNA replication, base excision repair, RNA transport, spliceosome and so on while down-regulated genes were related to valine, leucine and isoleucine degradation, fatty acid degradation, metabolic pathways, and protein processing in endoplasmic reticulum, etc. (Additional file 2: Figure S1).

### Overview of whole genome bisulfite sequencing

**Fig. 3:**
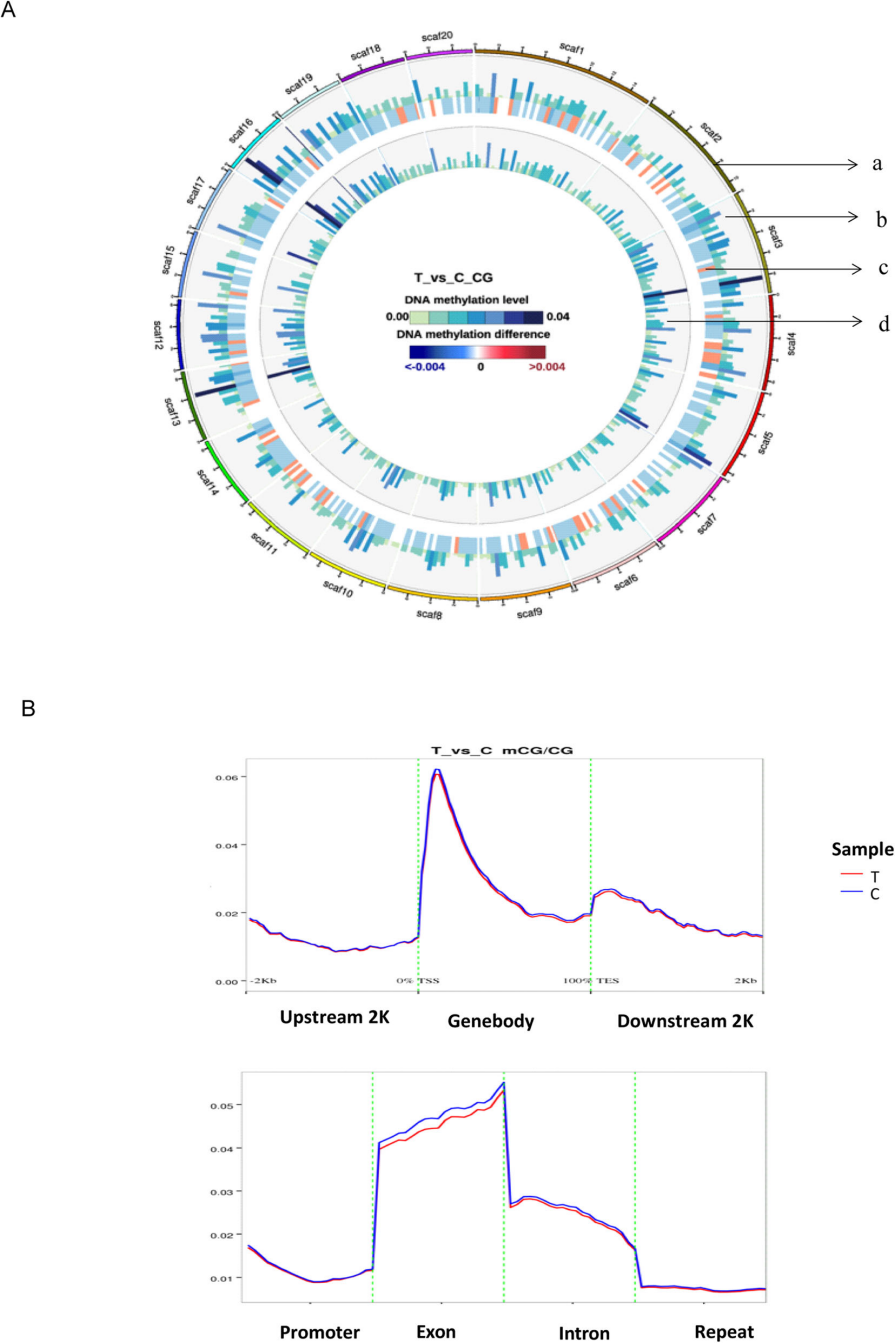
The global difference methylation level between T and C groups on CG site. **a** Circos images were used to show the different methylation levels between C group (normal midgut) and T group (BmNPV-infected midgut) at the genome-wide scale. Each circle from the outside to the inside depicts: (a) 20 scaffolds of *Bombyx mori* from the largest to the smallest size distributed in clockwise; (b) the methylation level of T group; (c) difference methylation level between T groups and C group; (d) the methyaltion level of C group. Color scale from green to blue represents DNA methylation level; Color scale from blue to red represents DNA methylation difference between two groups. **b** Distribution of DNA methylation levels of genes in T and Cgroup. The gene structure is defined by seven different features, denoted by the x-axis. The length of each feature was normalized and divided into equal numbers of bins

To evaluate epigenetic changes in insect cells caused by BmNPV infection, genome-wide DNA methylation profiles of BmNPV-infected midgut (T group) and normal midgut (C group) were performed at single-base resolution. Based on more than 99.8% bisulfite conversation rate and more than 10× mean sequencing coverage on cytosine site, we observed that about 0.185% of genomic cytosine was methylated, which was higher than 0.11% in the silk glands of silkworm (Xiang, 2010). The average methylation level at CG location was about 0.875%. The global difference methylation level between T group and C group was displayed by Circos software, which can visualize data in circular layout [28](Fig. 3a). Relatively, on scaffold 4 and 11, some methylated sites were shown hyper-methylation while on scaffold 5 and 19; a lot of hypo-methylated sites were observed (T vs. C). Furthermore, we comparatively analyzed the methylation level on different genomic functional regions between these two groups and the results revealed that in silkworm, DNA methylation was mostly targeted to gene bodies. It peaked in exon region and sharply decreased in intron region. Promoter region showed more methylation than the repeat region (Fig. 3b). Fractional methylation levels in exon region exhibited the tendency of hypo-methylation in BmNPV-infected midguts than in control midguts (Fig. 3b).

### Differential DNA methylation regions (DMRs) upon BmNPV infection

**Fig. 4:**
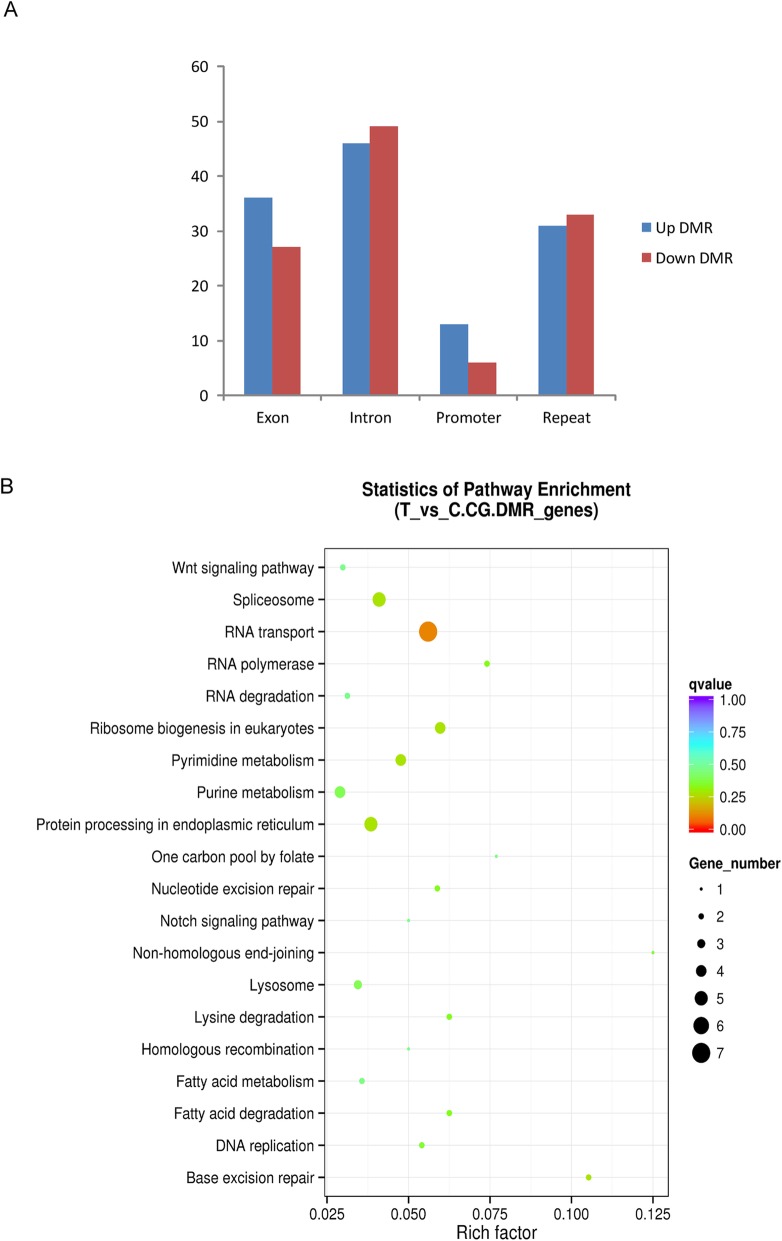
Differentially methylated genes associated with BmNPV infection. **a** The number of differentially methylated genes in different regions of the genome. **b** KEGG pathway enrichment of differentially methylated genes. RichFactor is the ratio of the number of differentially expressed genes in this pathway term to the number of all genes in this pathway term. Q-value is corrected for ranging from 0 to 1. Only the top 20 of the enriched pathway terms are displayed here

To explore the potential function of DNA methylation in response to BmNPV infection, we compared the DMR of the whole genome between BmNPV infected midgut and the control tissues. The results revealed that 241 DMRs were obtained with 177 located in the gene region. Among these, 126 DMRs were hyper-methylated and 115 were hypo-methylated (Additional file 1: Table S3). Further study revealed that DMRs are preference for the intron region (Fig. 4a). KEGG pathway analysis of 177 differential methylation genes (DMGs) revealed that DMGs were involved in pathways of spliceosome, RNA transport, protein processing in endoplasmic reticulum, etc. (Fig. 4b).

### Effects of DMRs on gene expression in silkworm challenged by BmNPV infection

**Fig. 5:**
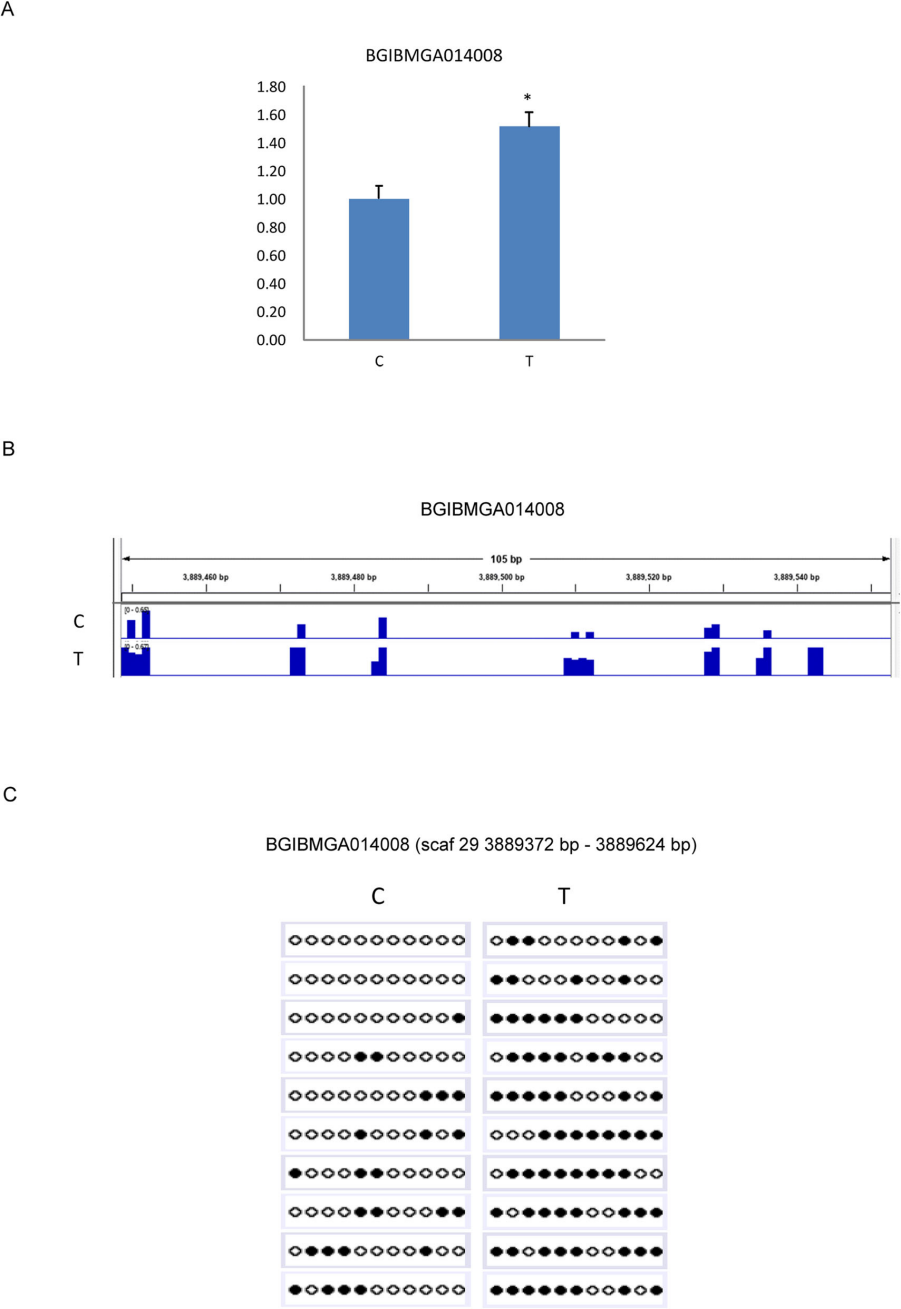
Validation of both different expression and different methylation of BGIBMGA014008 in response to BmNPV infection. **a** BGIBMGA014008 was identified to be differentially expressed upon BmNPV infection by qRT-PCR. RNA was extracted from midguts with or without BmNPV infection. Data represent the relative transcript levels of genes in the infected midguts compared to the control tissues from three independent samples. The error bars indicate standard deviations (*p < 0.05). C group represents samples from normal midguts and T group representssamples from BmNPV-infected midguts. **b** The visualized image of differential methylation of BGIBMGA014008was generated by Integrative Genomics Viewer (IGV). The location of blue bars in the images indicates the methylated sites and the height of blue bars represents the methylation levels. **c** Bisulfite sequencing validation of differential methylation of BGIBMGA014008in normal verse infected midgut. Targeted bisulfite sequencing validation of a region overlapping the DMR is indicated by the rectangle over the genome browser tracks. Dark circles indicate methylated and open circles mean unmethylated cytosines. Each row consists of a single sequenced clone

To investigate whether the variances in CG methylation upon BmNPV infection could alter gene expression, we comprehensively analyzed the data of DEGs and DMRs. As a result, we found 26 DMGs showing differential mRNA levels in the midgut of the infected silkworm (Table 1), suggesting that the expression levels of these genes involved in BmNPV infection may altered via variation in DNA methylation. Analysis of the distribution of DNA methylation on these genes revealed that CG methylation of 25 genes presented in the gene body, predominantly in the introns of genes, accounted for about 61.54% (Additional file 3: Figure S2). Interestingly, there were 2 genes (*BGIBMGA002246* and *BGIBMGA004695*), whose intron-exon boundaries were DNA methylated, indicating that DNA methylation may be associated with alternative splicing in *Bombyx mori* [29]. Furthermore, by qRT-PCR and BS-PCR, we validated the up-regulation (Fig. 5a) and hyper-methylation of *BGIBMGA014008* (Fig. 5b, c), encoding for structural maintenance of chromosomes protein in BmNPV-infected midgut.

**Table 1:** Gene informatin of both DEGs and DMRs in silkworm midguts with BmNPV infection

Gene ID	Fold changes (T vs. C)	*P*-value	Differentially methylated level (T vs. C)	Area statistic	Region	Annotation
BGIBMGA002246	3.175	1.2E-11	0.0602	21.29	exon/intron	Zinc finger, RING
BGIBMGA002591	2.182	7.8E-03	−0.1573	−57.81	intron	Serine/threonine-protein kinase, active site
BGIBMGA002694	1.997	2.6E-05	0.1536	177.73	intron	Nucleoporin Nup188
BGIBMGA002730	0.429	1.1E-08	−0.1845	−73.42	exon	Short-chain dehydrogenase
BGIBMGA004196	0.602	4.3E-02	0.1129	67.73	exon	Peptidase A22B, signal peptide peptidase
BGIBMGA004325	2.089	8.9E-03	0.1390	69.91	intron	
BGIBMGA004568	2.012	6.2E-05	0.1440	63.71	intron	RSBN1/Dpy-21
BGIBMGA004695	2.237	1.3E-05	0.1231	55.51	exon/intron	AT hook, DNA-binding motif
BGIBMGA004825	7.999	1.4E-43	0.0392	44.28	intron	DNA-directed DNA polymerase, family B, multifunctional domain
BGIBMGA004826	2.342	5.1E-03	0.1471	35.46	exon	DNA polymerase epsilon catalytic subunit
BGIBMGA004940	0.601	2.0E-03	−0.2389	−34.33	intron	E3 ubiquitin-protein ligase synoviolin/Hrd1
BGIBMGA005449	3.191	1.2E-15	−0.0526	−42.73	intron	DNA repair protein, RAD50, zinc hook
BGIBMGA005731	1.696	2.0E-03	0.0434	65.54	intron	Armadillo-like helical
BGIBMGA006028	1.810	4.2E-02	−0.1486	−28.80	intron	Mediator of RNA polymerase II transcription subunit 15
BGIBMGA007840	2.992	4.6E-10	−0.0734	−56.96	exon	Zinc finger, PHD-type, conserved site
BGIBMGA008783	1.760	1.4E-02	0.0985	49.17	intron	Zinc finger C2H2-type
BGIBMGA009018	0.661	1.3E-02	0.1037	94.04	exon	DHS-like NAD/FAD-binding domain
BGIBMGA009805	1.744	1.6E-02	0.2161	116.58	exon	Actin-like protein 6A
BGIBMGA009855	1.714	3.9E-02	−0.1710	−103.49	intron	PHD-finger 5A
BGIBMGA010559	0.597	4.0E-03	0.0458	55.06	intron	Acyltransferase ChoActase/COT/CPT
BGIBMGA011887	1.768	2.0E-02	−0.1481	−77.02	promoter	Dihydroorotate dehydrogenase domain
BGIBMGA011899	0.587	1.5E-03	0.1664	79.91	intron	Histidine triad (HIT) protein
BGIBMGA011924	3.062	6.7E-05	0.1600	29.70	exon	SAPAP family
BGIBMGA013820	0.519	4.1E-02	−0.0381	−74.62	intron	Potassium channel, inwardly rectifying, Kir, cytoplasmic
BGIBMGA013922	2.230	1.1E-03	0.0323	18.60	intron	
BGIBMGA014008	2.241	2.9E-06	0.1837	89.62	intron	Structural maintenance of chromosomes protein

### DNMT inhibition decreases the expression of antiapoptosis-related genes in *Bombyx mori*

**Fig. 6:**
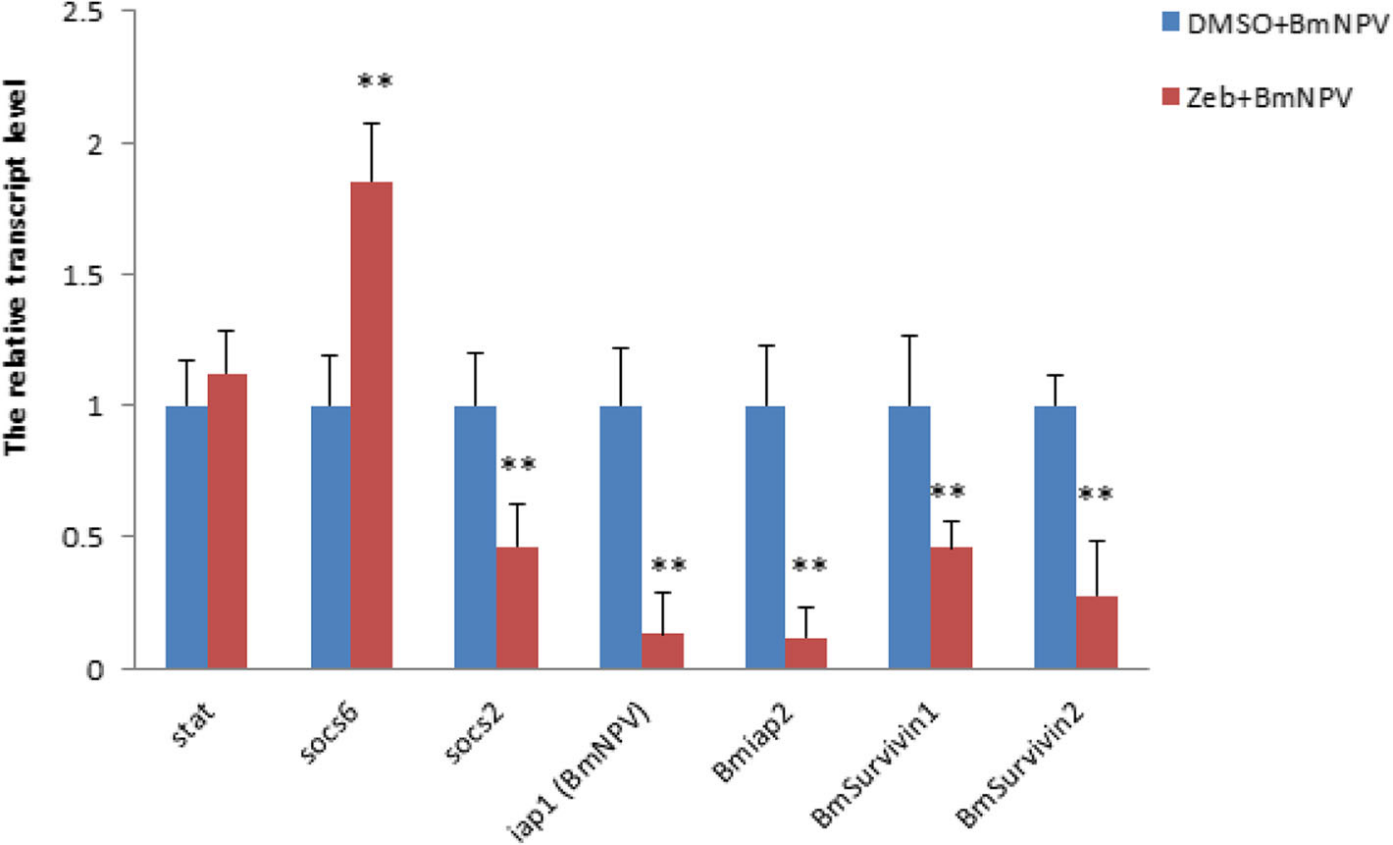
DNMT inhibition alters the expression of genes involving in antiapoptosis. RNAs were extracted from BmNPV-infected BmN cells treated with Zeb and DMSO, respectively and then transcribed into cDNA as template for RT-qPCR. The data were normalized using *BmGAPDH* and are represented as mean ± SD from three independent experiments. Relative expression levels were calculated using the 2^−ΔΔCt^ method(**p < 0.01)

To explore the potential mechanism of DNA methylation on regulating host immune response to BmNPV infection, we detected the expressed level of 7 genes, which are associated with JAK/STAT pathway (*stat, socs6 and socs2*) and anti-apoptosis (*iap1* from BmNPV*, Bmiap2, BmSurvivin1, BmSurvivin2*) between Zeb-treated cells and control cells. The results showed that the transcript level of *socs6* is up-regulated and *socs2* is down-regulated while that of *stat* has no significant change (Fig. 6). Interestingly, the expression levels of all the four genes related to anti-apoptosis are significantly down-regulated (Fig. 6), indicating that DNMT inhibition may promote the apoptosis of infected cells by suppressing the expression of anti-apoptosis genes both in virus and in *Bombyx mori*.

## Discussion

The role of epigenetic regulation of gene expression has been widely accepted in animal and plants with the change of environmental stressors, such as the invasion of pathogens. Recent studies have shown that inhibition of DNA methylation by chemicals such as 5-aza-2′-deoxycytidine significantly induced antitumor immune responses in colon and ovarian cancers [9, 10] and resulted in cell cycle arrest, p53-dependent apoptosis, and IFN signaling activation of HPV-positive HNC cells [30]. In this study, we found that inhibition of DNMT by Zeb could suppress BmNPV replication.

To explore whether the BmNPV infection can induce the variance on genomic DNA methylation of silkworm and whether DNA methylation could regulate the expression of genes associated with BmNPV infection, we performed transcriptome analyses and whole-genome bisulfite sequencing between BmNPV-infected midguts and control midguts of silkworm larvae. As a result, the characteristics of genomic DNA methylation in *Bombyx mori* are similar to previous studies on other insects and distinct from that of animal and plant. For insects, the general methylation level is much lower than that in mammals. At current, the cytosine methylation level of most insects is about 0 ~ 1%, while in mammals and birds’ ranges from 3 to 10%, fish and amphibians around 10%, and some plants up to 50% [28]. In most mammals, about 60 to 90% of all CpG dinucleotides are methylated. DNA methylation in vertebrates distributes in the entire genome [31] while due to the low level of methylation in insects, DNA methylation in insects is scattered throughout the genome and is mainly concentrated in gene body (introns and exons). Promoters and repeat region DNAs in insects are hypo-methylated [32]. In this study, as to *Bombyx mori*, it exhibits low levels of DNA methylation with about 0.185% and CG methylation is substantially enriched in gene bodies.

The function of DNA methylation on gene expression is well established, but the underlying mechanisms of this function are poorly understood. It is speculated that cytosine methylation can facilitate or suppress interaction with DNA-binding protein, change the stability of nucleosomes, affect the local chromatin structure and access of transcript factors to genomic DNA, and thus result in changes of gene expression [33, 34]. In this study, we found 26 DEGs showing differential methylated levels in the midgut of BmNPV-infected silkworm larvae with 17 hyper-methylated and 9 hypo-methylated, which demonstrated that variance in DNA methylation may lead to expression changes of those genes associated with BmNPV infection.

BGIBMGA014008 is a structural maintenance of chromosomes protein gene. Studies showed that structural maintenance of chromosomes 4 (SMC-4) is a chromosomal ATPase which plays an important role in regulating chromosome assembly and segregation. SMC-4 expression was significantly higher in colorectal cancer and was associated with tumorigenesis. Knockdown of SMC-4 significantly suppressed the proliferation of cancer cells and degraded its malignant degree [35]. In addition, SMC4 expression was significantly associated with tumor size, dedifferentiation, advanced stages and vascular invasion of the primary liver cancers [36]. In our study, BGIBMGA014008 was identified up-regulation and hypermethylation in the midgut after BmNPV infection (Fig. 5), indicating that BGIBMGA014008 may involve in silkworm cell multiplication and play roles in BmNPV infection.

Dihydroorotate dehydrogenase (Dhodh), catalyzing the oxidation of dihydroorotate to orotate, is the fourth enzyme of pyrimidine synthesis in the de novo pyrimidine biosynthetic pathway [37]. It is reported that down-regulation of BmDhodh inhibits cell growth and the endomitotic DNA replication in silk gland cells [38]. In our study, we observed the increased mRNA level and hypo-methylation in promoter region of BmDhodh (BGIBMGA011887). Generally, promoter methylation represses gene expression [39]. We speculated that hypomethylation in the promoter region of BmDhodh activates BmDhodh expression, which is benefit for silkworm cell replication, and thus facilitates BmNPV proliferation.

Nuclear import and export of viral RNA and proteins are critical to the replication cycle of viruses. Regulation of this process is paramount to processes such as cell division and differentiation, but is also critically important for viral replication and pathogenesis [40]. Nucleocytoplasmic transport is mediated by nuclear pore complexes (NPCs), which are embedded in the nuclear envelope [41]. Nup188, one of the nucleoporins can regulate chromosome segregation, promote chromosome alignment, confine the passage of membrane proteins and is thus crucial for the homeostasis of the different nuclear membranes [42, 43]. In this study, both the mRNA transcript level of Nup188 (BGIBMGA002694) and DNA methylation level in the intron of Nup188 were significantly enhanced in BmNPV infected midgut, suggesting that DNA methylation may have impact on nucleoporins expression and thus affect the replication of BmNPV.

Other interesting genes, such as Rad50 (BGIBMGA005449), which is associated with DNA repair functions [44–46] and the histidine triad protein gene (BGIBMGA011899), which plays roles in transcription, signal transduction and many peripheral and central nervous system diseases [47] are also shown different mRNA transcript level and DNA methylated level upon BmNPV infection (Table 1).

Recent studies have revealed that gene expression regulation by DNA methylation may play a critical role in arms races between viruses and their hosts [48]. To evade detection and restriction by the host immune response, viruses also employ various mechanisms to control gene expression related to immunity, including hijacking epigenetic machinery [49, 50]. Studies on virus-driven dysregulation of host immune-related gene expression through DNA methylation present a novel viral mechanism to inhibit immune responses [51]. Many DNA and RNA virus could utilize this mechanism to down-regulates expression of host immune-related genes [6, 52, 53].

Previous reports revealed that BmNPV infection could activate the JAK/STAT signaling pathway and has slight effects on Imd and Toll signaling pathways [54–56]. The JAK signal transducer and STAT signaling pathway is an important regulator of cell proliferation, differentiation, survival, apoptosis and immune response [57]. Therefore, to explore whether DNA methylation could affect the JAK/STAT pathway, we detected the expression of three key genes involved in this pathway and we found that after inhibition of DNMT, two genes, which are negative regulators of the JAK/STAT signaling pathway [58], displayed the opposite expression pattern with up-regulation of *socs6* and down-regulation of *socs2*. Another key gene, *stat* has no significant expression changes (Fig. 6), indicating that DNA methylation may have no obvious effects on JAK/STAT pathway.

Apoptosis as an important immune response plays a critical role in the antiviral defense in insects [59]. Inhibitor of apoptosis (IAP) protein family have demonstrated functions in both apoptosis and innate immunity [60]. Both BmIAP and Baculoviruses encode IAP had an inhibitory effect on apoptosis in insects [61, 62]. Survivin, another apoptosis inhibitor has been confirmed to be an essential regulator of cell division [63]. In our study, we found four genes belong to inhibitor of apoptosis family were all decreased their expression (Fig. 6), suggesting that DNMT1 inhibition may lead to promote the apoptosis of infected cells and consequently suppress the BmNPV proliferation.

## Conclusions

In summary, based on parallel analyses of global gene expression and the cellular methylome upon BmNPV infection, our results suggest that alterations in DNA methylation status may have effects on the expression of genes associated with BmNPV infection. Inhibition of DNMT in silkworm cells could induce the apoptosis of infected cells. Our results may provide a new idea and research direction for the molecular mechanism of the interaction between silkworm and viruses.

## Methods

### Cell line, silkworm strain and virus inoculation

The *Bombyx mori* cell line BmN, which was kindly gifted by Dr. Xudong Tang from Silkworm Pathology Laboratory in Jiangsu University of Science and Technology, was cultured at 27 °C in TC-100 medium (United States Biological, USA) supplemented with 10% (V/V) fetal bovine serum (FBS) (Gibco, USA) in 6-well plates to a confluency of about 70%. BmN cells per well were infected with recombinant BmNPV BVs containing an *egfp* marker gene at a MOI of 5. The P50 silkworm strain was reared at room temperature and under a photoperiod of 12 h light and 12 h dark until the fourth molt. For viral inoculation, 2 μL BmNPV viral stock including recombinant BmNPV BVs was injected into each larva through intersegmental membrane. The control uninfected larvae were injected with 2 μL 0.9% NaC1 solution. The infected group and the uninfected group had 3 replicates respectively and each replicate was pooled with 5 larvae.

### DNMT inhibitor and cellular cytotoxicity assay (MTT)

BmN cells were seeded in 96 well plates at a density of about 5000 cells per well and were treated with different concentrations of DNMT inhibitor, Zeb (MedChemExpress) for 12 h. 10 μl MTT (5 mg/ml, Sigma) was added to each well at 37 °C in 5% CO_2_ for 4 h. Later, the medium was removed carefully without disturbing the formazan crystals and 150 ml of dimethyl sulfoxide (DMSO) was added followed by incubation at 37 °C for 15 min. The absorbance of the suspension was measured at 490 nm. The percentages of metabolically active cells were compared with the percentage of control cells of the same culture plate. Cellular cytotoxicity was determined in duplicate and each experiment was repeated three times independently.

### Quantitative real-time PCR analysis

Relative quantitative real-time PCR (qRT-PCR) as descried in our previous study was performed to detect the expression of silkworm gene [64, 65]. As to BmNPV gene *lef-3* (GeneID: 1488686) and *ie-1* (GeneID: 1488755), absolute qRT-PCR was carried out as in our previous report [64]. PCR reactions were run with a thermal cycling at 95 °C for 30 s followed by 40 cycles of 95 °C for 5 s, 60 °C for 31 s. Following the amplification, melting curves were constructed. Three independent experiments with three technical replicates were analyzed. All data were represented by the mean ± SD. The unpaired Student’s t test was used to compare the difference in means. *P* value < 0.05 was set for statistically significant. The sequences of the primers were listed in Additional file 1: Table S4.

### Western blot assay

Western blotting assay was performed as descried in our previous study [64]. Briefly, a total of 30 μg protein sample from cells was loaded on each lane and separated on SDS-PAGE before transferred onto a nitrocellulose membrane. The membrane was incubated with rabbit VP39 (1:2000). The same blots were re-probed with ɑ-Tubulin ployclonal antibody (1:2000; Sigma, USA) to confirm equal loading of samples. The secondary antibody of VP39 and ɑ-tubulin is HRP labeled goat anti-rabbit IgG (1:1000; Beyotime, China).

### Transcriptome analysis

RNA-Seq experiment with three biological replicates was performed by Novogene Bioinformatics Technology Co., LTD (Beijing, China). The process is described briefly as follows: total RNA was extracted by using the Trizol reagent (Invitrogen, USA) and RNA integrity was assessed using the RNA Nano 6000 Assay Kit of the Bioanalyzer 2100 system (Agilent Technologies, CA, USA). Sequencing libraries were generated using NEBNext® Ultra™ RNA Library Prep Kit for Illumina® (NEB, USA) following manufacturer’s recommendations. The library fragments of preferentially 250~300 bp in length were purified with AMPure XP system (Beckman Coulter, Beverly, USA). Then, the fragments were ligated to sequencing adaptors and enriched by PCR amplification. The libraries were sequenced on an Illumina Hiseq platform after library quality was assessed on the Agilent Bioanalyzer 2100 system.

Clean reads, which were obtained by removing reads containing adapter, reads containing ploy-N and low quality reads from raw data were mapped to silkworm genomic database(*B. mori* assembly ASM15162v1) using Hisat2 v2.0.4. Differentially expressed genes were identified by the DESeq R package (1.18.0). The resulting *P*-values were adjusted using the Benjamini and Hochberg’s approach for controlling the false discovery rate. Genes with an adjusted P-value < 0.05 and fold change ≥2 were assigned as differentially expressed. Gene Ontology (GO) enrichment analysis of differentially expressed genes was performed by the GOseq R package. GO terms with corrected P-value less than 0.05 were considered significantly enriched by differential expressed genes. KOBAS software was used to test the statistical enrichment of differential expression genes in KEGG pathways (http://www.genome.jp/kegg/) [66].

### Whole genome bisulfite sequencing

For whole-genome bisulfite sequencing, three biological replicates were performed. A total amount of 5.2 mg genomic DNA were fragmented by sonication to 200-300 bp, followed by end repair, adenylation and ligating methylated adaptors. Then these DNA fragments were treated twice with bisulfite using EZ DNA Methylation-Gold™ Kit (Zymo Research) before PCR amplification. Finally, sequencing was performed on an Illumina Hiseq 4000 platform and 125 bp/150 bp paired-end reads (raw reads) were generated. The clean reads were obtained from raw reads after pre-processed through Trimmomatic (Trimmomatic-0.36) software and all filtering steps.

Bismark software (version 0.16.3) [67] was used to perform alignments of bisulfite-treated reads to silkworm genome (*B. mori* assembly ASM15162v1). Firstly, silkworm genome was changed into bisulfite-converted version (C-to-T and G-to-A) followed by indexing using bowtie2 [68]. Then, sequence reads were changed into bisulfite-converted versions before aligned to converted genome in a directional manner. The unique best alignment reads are then compared to the normal genomic sequence and the methylation level of all cytosine in the reads is evaluated.

The results of methylation extractor were transformed into bigWig format for visualization using IGV browser. In order to calculate the methylation level of the sequence, we divided the sequence into multiple bins with bin size is 10 kb. The sum of methylated and unmethylated read counts in each window was calculated. Methylation level (ML) for each C site shows the fraction of methylated Cs, and is defined as:

$$\boldsymbol{ML}\left(\mathbf{C}\right)=\frac{\boldsymbol{reads}\left(\mathbf{mC}\right)}{\boldsymbol{reads}\left(\mathbf{mC}\right)+\mathbf{reads}\left(\mathbf{C}\right)}$$

Calculated ML was further corrected based on bisulfite non-conversion rate and sequencing coverage depth on a certain site. To obtain the accurate mC sites, threshold values were set up as follows: sequencing depth is≥5, q-value is≤0.01.

### Differentially methylated analysis

Differentially methylated regions (DMRs) were identified using the DSS software (version DSS_2.12.0) [8, 65, 69] . The core of DSS is a new dispersion shrinkage method for estimating the dispersion parameter from Gamma-Poisson or Beta-Binomial distributions. Putative DMRs were identified based on the following standards and parameters: (1) in the smoothing process, the smoothing distance is 200 bp (parameter: smoothing = TRUE, smoothing.span = 200, delta = 0); (2) the proportion of the difference sites with *P* value less than 1e-05 was greater than 50% of the region (parameter: p.threshold = 1e-05; pct.sig = 0.5); (3) the number of the sites was greater than 3, and the length was greater than 50 (parameter: minlen = 50; minCG = 3); (4) when the distance between two DMR is less than 100 bp, the two regions are merged (parameter: dis.merge = 100). GO and KEGG pathway analysis of genes containing DMRs were performed as described in the “Transcriptome analysis” section.

### Bisulfite-PCR validation of DMRs

Genomic DNA from BmNPV-infected and control tissues was extracted and treated with bisulfite as described above. Bisulfite converted DNA was uses for PCR amplification with ZymoTaq^TN^ Preix DNA Polymerase (ZYMO RESEARCH, America). Nested primers for BS-PCR were designed using the online MethPrimer program (Additional file 1: Table S4). PCR products were purified and cloned into the pMD19-T vector (TaKaRa, Japan). Ten different clones for each group were sent to Sangon Biotech Co., Ltd. (Shanghai, China) for sequencing. DNA methylation of individual CG sites were analyzed using Quma software (http://quma.cdb.riken.jp/).

## Supplementary information

**Additional file 1: Table S1.** Summary of the sequenced reads. **Table S2.** Differentially expressed genes of silkworm midguts upon BmNPV infection. **Table S3.** Differential DNA methylation regions of silkworm midguts upon BmNPV infection. **Table S4.** Sequences of primers for qRT-PCR and BS-PCR.

**Additional file 2: Figure S1.** KEGG pathway enrichment of differentially expressed genes following BmNPV infection.

**Additional file 3: Figure S2.** The number of both differentially methylated genes and differentially expressed genes in different regions of genome.

## Data Availability

The raw data in this study are deposited in the NCBI Sequence Read Archive database (http://www.ncbi.nlm.nih.gov/sra/) under the accession number PRJNA541379.

## Abbreviations

BmNPV: *Bombyx mori* nucleopolyhedrosis virus
DEGs: Differentially expressed genes
DMGs: Differential methylation genes
DMRs: Differential DNA methylation regions
DMSO: Dimethyl sulfoxide
DNMT: DNA methyltransferase
IAP: Inhibitor of apoptosis
WGBS: Whole genome bisulfite sequencing
Zeb: Zebularine
