# Supplementary material for: DNA methylomes and transcriptomes analysis reveal implication of host DNA methylation machinery in BmNPV proliferation in Bombyx mori
Source: BMC Genomics. 2019 Oct 15;20:736. doi: 10.1186/s12864-019-6146-7 (PMC6792228; doi:10.1186/s12864-019-6146-7)
Supplement: Supplementary file 2 — Additional file 2: Figure S1. KEGG pathway enrichment of differentially expressed genes following BmNPV infection. [file 12864_2019_6146_MOESM2_ESM.pdf]

A

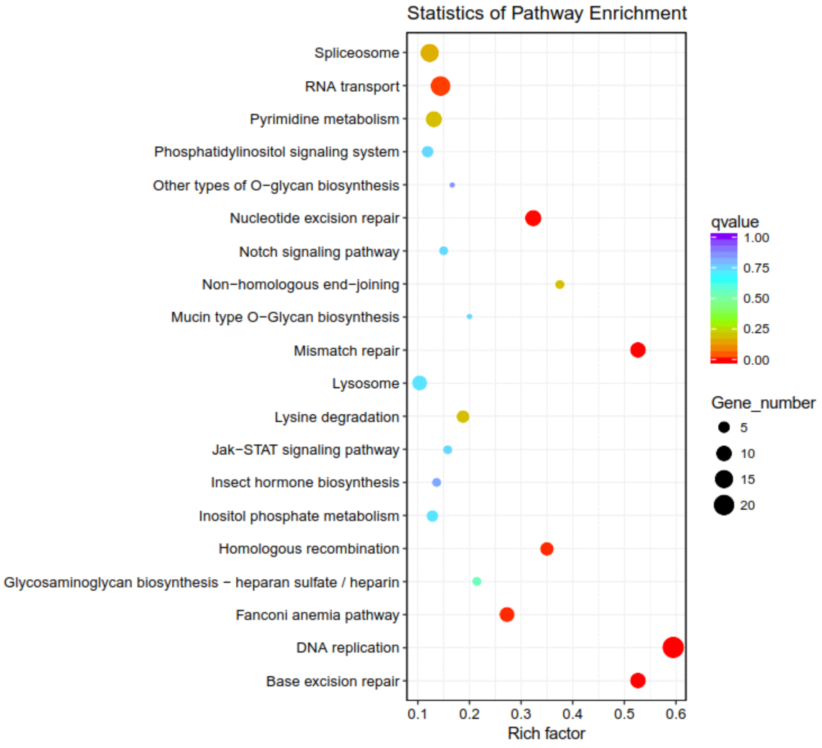

B

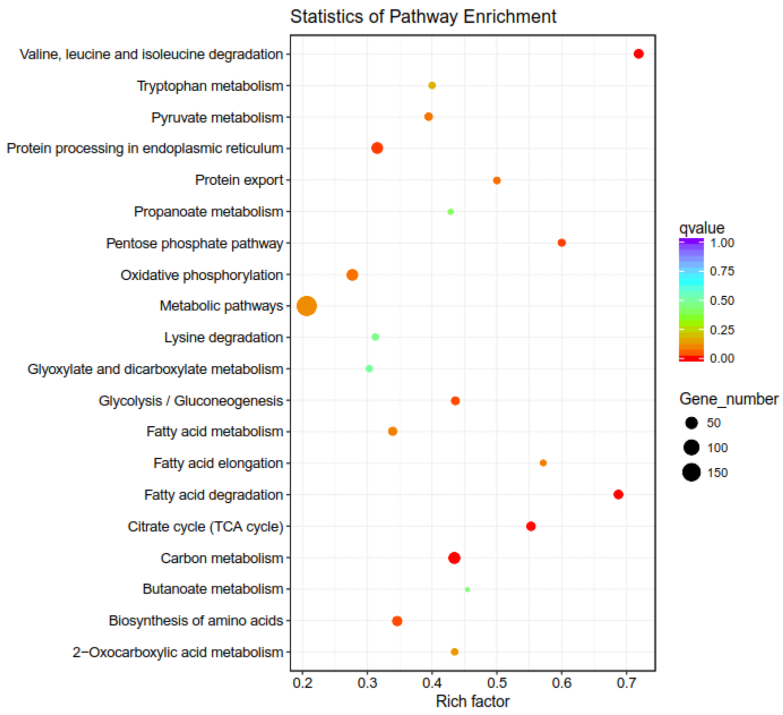

Figure S1 KEGG pathway enrichment of differentially expressed genes following BmNPV infection. (A) Up-regulated genes in infected midguts. **(B)** Down-regulated genes in infected midguts.
