# Supplementary material for: DNA methylomes and transcriptomes analysis reveal implication of host DNA methylation machinery in BmNPV proliferation in Bombyx mori
Source: BMC Genomics. 2019 Oct 15;20:736. doi: 10.1186/s12864-019-6146-7 (PMC6792228; doi:10.1186/s12864-019-6146-7)
Supplement: Supplementary file 3 — Additional file 3: Figure S2. The number of both differentially methylated genes and differentially expressed genes in different regions of genome. [file 12864_2019_6146_MOESM3_ESM.pdf]

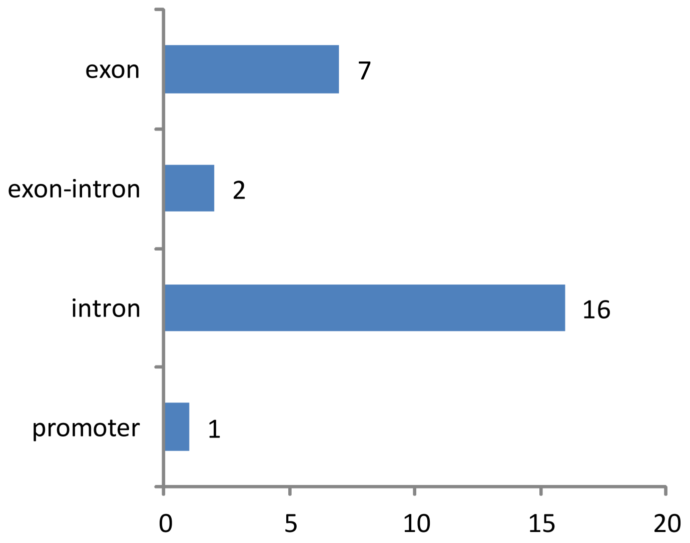

Figure S2 **The number of both differentially methylated genes and differentially expressed genes in different regions of the genome**.
